# Rv1288, a Two Domain, Cell Wall Anchored, Nutrient Stress Inducible Carboxyl-Esterase of *Mycobacterium tuberculosis*, Modulates Cell Wall Lipid

**DOI:** 10.3389/fcimb.2018.00421

**Published:** 2018-12-03

**Authors:** Pratibha Maan, Arbind Kumar, Jashandeep Kaur, Jagdeep Kaur

**Affiliations:** Department of Biotechnology, Panjab University, Chandigarh, India

**Keywords:** *Mycobacterium tuberculosis*, esterase, cell wall, nutrient starvation, TDM content, peptidoglycan binding

## Abstract

Rv1288, a conserved hypothetical protein of *M. tuberculosis (M.tb)*, was recently characterized as two-domain esterase enzyme by *in silico* study. In the present study, Rv1288 and its domains (Est and Lyt) were cloned individually from *M.tb* into *E. coli* for expression and purification. The purified rRv1288 and rEst proteins exhibited lipolytic activity with medium chain length esters as optimum substrates, while Lyt domain did not show enzymatic activity. However, presence of Lyt domain resulted in enhanced rate of protein aggregation at higher temperature. Both rRv1288 and rEst followed the similar patterns of substrate specificity, temperature and pH activity. Site directed mutagenesis confirmed the Ser-294, Asp-391 and His-425 as catalytic site residues. Rv1288 was found to be present in cell wall fraction of *M.tb* H37Ra. Peptidoglycan binding activity of Rv1288 and its domains demonstrated that the Lyt domain is essential for anchoring protein to the cell wall. Expression of *rv1288* was up regulated in *M.tb* under nutrient starved condition. Over expression of *rv1288* in surrogate host *M. smegmatis* led to change in colony morphology, enhanced pellicle and aggregate formation that might be linked with the changed lipid composition of bacterial cell wall. Cell wall of *M. smegmatis* expressing *rv1288* had higher amount of lipids, with a significant increase in trehalose dimycolate content. Rv1288 also leads to increase in drug resistance of *M. smegmatis*. Rv1288 also enhanced the intracellular survival of *M. smegmatis* in Raw264.7 cell line. Overall, this study suggested that Rv1288, a cell wall localized carboxyl hydrolase with mycolyl-transferase activity, modulated the cell wall lipids to favor the survival of bacteria under stress condition.

## Introduction

Tuberculosis (TB) plagued humankind since the beginning of medical history. Even today, it is the leading killer of human beings globally from any infectious disease. According to WHO global tuberculosis report 2017, approximately 6.3 million new cases and 1.6 million deaths from TB were reported in 2016, across the world (WHO, 2016). The gravity of the situation had worsened due to poorly understood biology, pathogenesis and long dormancy stage of causative organism *Mycobacterium tuberculosis (M.tb)*, undiagnosed primary and re-infection, reasonably asymptomatic condition, limited anti-TB drugs, long duration treatment resulting in poor compliance by patients, the emergence of drug-resistant strains and co-infection with HIV (Ford et al., 2015; Petersen et al., 2017; Queiroz and Riley, 2017). Hence new, effective and cheap drugs active against active as well as latent TB, compatible with antiretroviral therapy, are the need of the hour (Mahamed et al., 2017). Under these circumstances identification of new drug targets, which might be explored to develop new anti TB drugs, are urgently needed.

Lipids and lipid metabolism played significant role in infection, intracellular survival and pathogenicity of *M.tb*. Cell wall lipids were reported to interact with host resulting in modulation of metabolism and immune response, regulation of transport of drugs, nutrients and other molecules. During infection, *M.tb* stored lipids in the form of triacylglycerol, and hydrolyzed them for nutrition and energy requirements during latent and reactivation stage (Lovewell et al., 2016; Barisch and Soldati, 2017; Queiroz and Riley, 2017; Gago et al., 2018). Approximately 6% of *M.tb* genome encoded for the lipid metabolism enzymes (Lew et al., 2011). *M.tb* had 26 genes, encoding for putative lipases (Lip A to Lip Z), classified as lip gene family (Cole et al., 1998). However, other than these, many other proteins, possessing the typical characteristics of lipase/esterase enzymes (α/β serine hydrolase fold and conserved penta-peptide sequence) were identified bioinformatically. Though α/β serine hydrolase fold is possessed by many other enzyme families such as acetylcholineesterase, thioesterase, proline iminopeptidase/oligopeptidase, serine carboxylpeptidase, eposide hydrolase and many more (Holmquist, 2000), several of these proteins demonstrated lipolytic activity contrary to their annotated function (Lun and Bishai, 2007; Anand et al., 2011; Kumar et al., 2017c). The possible role of several lipases/esterases in the life of *M.tb* had been investigated extensively till date. These were involved in the various functions such as synthesis and maintenance of *M.tb* unique cell envelop, degradation of host cell lipids, xenobiotic degradation, toxic epoxide degradation, evasion and modulation of host immune system, elicit humoral response, dentritic cell maturation and antigen presentation, adaptation during adverse conditions like dormancy and acidification (Deb et al., 2006; Xu et al., 2010; Madan-Lala et al., 2014; Singh et al., 2014c, 2016b; Jadeja et al., 2016; Barisch and Soldati, 2017; Chownk et al., 2017; Johnson, 2017; Kaur et al., 2017a; Kumar et al., 2017b; Vemula et al., 2018). Such variety of functions were possible due to the fact that, these mycobacterial lipolytic enzymes possess many additional activities also like β lactamase, Mycolyltransferase, TDM hydrolase, Eposide Hydrolase, Phospholipase, etc. (Zhang et al., 2005; Singh et al., 2016a; Chownk et al., 2017; Queiroz and Riley, 2017). One such activity is mycolyltransferase and TDM hydrolase activity. They are the most well-characterized member of mycobacterial alpha/beta hydrolases, both biochemically and genetically. Yang et al. (2014) reported Rv3451 as TDM hydrolase, which remodels the *M.tb* cell wall to enhance the nutrient flux, and increased its intracellular survival in MyD88-/- mice (Yang et al., 2014). A cutinase like serine carboxylesterase Msmeg_1529 acts as TDM hydrolase in *M. smegmatis* (Ojha et al., 2010). Therefore, these enzymes could be used as drug targets for developing new anti-mycobacterial drugs (Gago et al., 2018).

Approximately 4,000 genes were identified in *M.tb* genome, but still around 1,100 genes had unknown function and were classified under unknown or conserved hypothetical proteins category. Some of these hypothetical genes were also presumed to encode for lipolytic enzymes (Kumar et al., 2017d). Recently several studies were carried out to elucidate the role of such hypothetical proteins such as Rv2224c (Lun and Bishai, 2007; Rengarajan et al., 2008; Naffin-Olivos et al., 2017) and Rv0774c (Kumar et al., 2017c,e). However, lots of information was still buried in these hypothetical proteins. Therefore, to completely understand the physiology of *M. tb*, there is a need to find out the role of these unknown/uncharacterized gene products.

One such protein Rv1288, a 456 amino acid protein, was categorized as conserved hypothetical protein with probable protease activity in tuberculist database. This protein shared 26–31% sequence identity with the Antigen 85 complex (FbpA, FbpB, and FbpC) (Takayama et al., 2005). Homologous proteins of Rv1288 were found in other mycobacterium species such as *M. smegmatis, M. bovis, M. avium, and M. marinum* based on sequence alignment analysis. Rv1288 was composed of two domains, first LytE domain at N-terminal, consisted of three consecutive LysM motifs and second esterase domain at C- terminal belongs to esterase D family (Doerks et al., 2012). Kumar et al., performed the bioinformatics analysis by homology modeling and molecular docking to study the probable role of this protein in *M.tb*. It was suggested as a probable lipolytic enzyme by *in silico* experiments (Takayama et al., 2005; Kumar et al., 2017a). The LytE domain was predicted to be responsible for the binding of this protein with mycobacterial cell wall. Esterase domain showed the presence of HGGG tetrapeptide motif, GxSxG pentapeptide sequence and S294, D391 and H425 as catalytic triad. Further docking studies predicted the high binding affinity of serine hydrolase inhibitors such as THL and PMSF with this enzyme (Kumar et al., 2017a). However, all these properties were annotated on the basis of bioinformatics and prediction methods and needed to be validated by wet lab experiments. Therefore, in the present study, full length Rv1288 and its domains were cloned and expressed in *E. coli* in order to purify and characterized them. We analyzed the expression of Rv1288 under different stress conditions. Effect of Rv1288 on different cell surface properties was also explored to elucidate the functional role of Rv1288 in physiology of *M. tb*.

## Materials and methods

### Reagents, chemicals, bacterial strains, and vectors

All reagents and chemicals were purchased from Hi-Media laboratories (India), and Sigma- Aldrich (USA). All the bacterial strains were procured from IMTECH-MTCC, Chandigarh. *E. coli* DH5α and *E. coli* BL21 DE3 strains were grown in Luria Broth (LB media) at 37°C and 180 rpm. LB media supplemented with kanamycin (30 μg/ml) and chloramphenicol (35 μg/ml) antibiotics was used for growth of recombinant strains of *E. coli*. *M.tb* H37Ra and *M. smegmatis* mc^2^155 were grown in M7H9 media (Sigma Aldrich, USA) supplemented with 1% glycerol, 0.05% Tween-80 and 10% Oleic acid- albumin-dextrose-catalase (OADC) (BD Bioscience), at 37°C and 180 rpm. For growth of *M. smegmatis* recombinant strains, kanamycin and hygromycin were added to M7H9 media at a concentration of 50 μg/ml and 100 μg/ml.

### Expression of *rv1288* in *M. tuberculosis* H37Ra under various stress conditions

*M.tb* H37Ra culture was inoculated in M7H9 media and grown at 37°C, 180 rpm up to mid log phase. Culture was centrifuged at 4,000 rpm for 10 min. Cell pellet was washed twice with 1X PBS (pH 7.4), re-suspended in stress media, such as acidic stress (M7H9 media, pH adjusted to 4.5 with HCl), nutrient stress (1X PBS, pH 7.4) and iron stress (using iron deficient media) (Kumar et al., 2017e) along with control culture (M7H9 media). All the samples were incubated at 37°C for 6 h. Total RNA was extracted from the samples using TRIzol Reagent (Sigma Aldrich, USA) according to manufacturer's guidelines along with cryogrinding. cDNA was prepared from equal amount of RNA of all samples by using Revert Aid first strand synthesis kit (Thermofisher, USA). Quantitative real time PCR (qRT-PCR) (Applied Biosystem® Step One^TM^ real time PCR) was performed to quantify the expression of *rv1288* in each sample using SYBR Green/ROX qPCR master mix (Fermantas). *16S rRNA* was used as an internal control in each set of reactions. Fold change in expression in different samples were calculated by 2^−ΔΔ*Ct*^. *rv3203, rv3097c*, and *rv560c* were used as positive control for acidic, nutritive and iron stress conditions, respectively (Rodriguez et al., 2002; Deb et al., 2006; Singh et al., 2014b). The specificity of results was validated by melting curve analysis.

### Cloning, expression, and purification of *rv1288, est*, and *lyt* domain

For cloning of *rv1288*, its domains *est* and *lyt* gene sequences were amplified from genomic DNA of *M.tb* H37Rv (kind gift from Dr. U.D. Gupta, JALMA, Agra, India) using designated set of primers (Table S1). Amplified products were excised and eluted from the gel. PCR product and pET28a expression vector were double digested with restriction enzyme (BamHI and HindIII. Digested gene fragments were ligated with vector by T4 DNA ligase (Invitrogen, USA). Ligated products were used to transform *E. coli* DH5α competent cells. Transformed cells were confirmed by sequencing (Xcelris Labs Ltd., India). Positive recombinant plasmids were transformed into the *E. coli* BL21 DE3 cells. All the cultures were grown overnight in 10 ml LB medium containing kanamycin. These overnight grown cultures were used to inoculate 250 ml of LB media. Bacterial cells were grown at 37°C, 180 rpm, until absorbance (600 nm) reached 0.4–0.6 i.e., mid log phase. All the cultures were induced with IPTG (0.05 mM) at 15°C, 180 rpm for 16–20 h. Cultures were centrifuged at 10,000 rpm for 20 min at 4°C. Cell pellet were suspended in 30 ml of cell lysis buffer (50 mM Sodium phosphate buffer, pH 8.0, 150 mM NaCl, 0.2% (v/v) triton-X 100). Cell lysates were further subjected to sonication (21% amplitude, 10 sec on and off pulse) for 10 min with intermittent cooling by Misonix Sonicator (Qsonia, USA). Cell debris were removed by centrifugation at 7,000 rpm for 20 min. Ni NTA column was equilibrated with equilibration buffer [sodium phosphate buffer (50 mM, pH 8.0), NaCl (300 mM), imidazole (20 mM)]. After equilibration, protein (supernatant collected by centrifugation) was passed through the column. Unbound or loosely bound proteins were washed from the column by passing 10 volume wash buffer [sodium phosphate buffer (50 mM, pH 8.0), NaCl (50 mM), imidazole (50 mM)]. Elution of bound protein was carried out by elution buffer [sodium phosphate buffer (50 mM, pH 8.0), NaCl (300 mM), imidazole (300 mM)] in 1 ml protein fractions. Absorbance at 280 nm was measured to find out the amount of protein in each fraction. All the fractions containing protein were pooled and dialyzed overnight in 50 mM phosphate buffer. The integrity and purity of protein was analyzed on 10% SDS-PAGE. Protein concentration was estimated by Bicinchoninic acid (BCA) method (kit by Banglore Genei, India).

### Skimmed milk plate assay

Skimmed milk plate assay were used for the qualitative determination of protease activity.

Skimmed milk (2%) agar plates were prepared in 50 mM sodium phosphate buffer (pH 8.0) and wells were punched (Brown and Foster, 1970; Drake and Montie, 1988). Hundred microliter of enzyme (50 μg/ml) and control buffer (100 μl) were added to the wells. Plates were incubated at 37°C for overnight. Trypsin (50 μg/ml) was used as positive control. Zone of hydrolysis around the well would indicate the protease activity of test protein.

### Tributyrin plate assay

The esterase activity of proteins was investigated by tributyrin plate assay method. Tributyrin emulsion (1%) agar plates were prepared in 50 mM phosphate buffer (pH 8.0). Assay was performed by adding 100 μl each of enzymes (50 μg/ml) and buffer as control in the wells. Plates were incubated at 37°C for overnight for developing zone of clearance.

### Enzyme assay

Enzymatic assays were carried out using *p*NP (*para* nitro phenyl)- derivative esters with little modifications. Reaction mixture was prepared by adding 700 μl sodium phosphate buffer (50 mM, pH 8.0), 100 μl protein (50 μg/ml), 100 μl sodium deoxycholate (10 mM), and 100 μl substrate (2 mM, *p*NP ester). Blank reaction mixture contained all components except for the protein. The reaction mix was incubated for 15 min at optimum temperature. After incubation 250 μl of Na_2_CO_3_ (0.1 M) was added to inhibit the reaction. Hydrolysis product was estimated by measuring absorbance at 420 nm was measured using in UV/Vis spectrophotometer (GE Healthcare, UK). One unit of enzyme activity was defined as the amount of enzyme, required to release 1 μmole of *para-*nitrophenol from substrate in 1 min under standard lipolytic enzyme assay conditions. Specific activity was expressed in U/mg of protein.

### Substrate specificity

The substrate specificity of rRv1288 and rEst proteins was determined by using different carbon chain length (C2, C4, C8, C10, C12, C14, C16, and C18) *p*NP esters as substrate in standard enzyme assay conditions. Stock solutions of substrates were prepared in absolute ethanol (10 mM stock).

### Biochemical characterization of rRv1288 and rEst

To determine the optimum temperature for enzyme activity, reaction mixture was incubated at temperature ranging from 20 to 80°C. To study the effect of pH, enzyme assay was performed in different pH buffers (pH 4.0 to 12.0) (Kumar et al., 2017e) and reaction mix was incubated at optimum temperature. Relative enzyme activity at various temperature/ pH was calculated by taking the maximum enzyme activity as 100%.

To find out the effect of temperature on stability of proteins, purified proteins (50 μg/ml) were incubated at different temperatures (20–80°C) for 1 h, and the cooled on ice for 10 min. Similarly, purified proteins were incubated in different pH buffer (pH 4.0 to 12.0) for 1 h at room temperature (RT). After incubation time, standard lipolytic enzyme assay was performed at optimum temperature and ph. Residual activity of enzymes was calculated by taking activity of un-incubated enzyme as 100%.

Enzyme assay was performed with different concentrations of *p*NP- caprylate (0.1–1 mM) to study the effect of substrate on rate of enzyme reaction. Lineweaver-Burk plot was drawn to calculate the Michaelis–Menten constant (*K*_m_), maximum velocity for the reaction (*V*_max_), *k*_*cat*_ and *k*_*cat*_*/K*_*m*_.

### Effect of phenylmethanesulfonylfluride (PMSF)

The purified rRv1288 and rEst proteins were incubated with PMSF (5 mM) at RT for 1 h. Standard enzyme assay was performed and residual enzyme activity of incubated proteins was calculated as compared to control (untreated proteins). The reaction mix without enzyme, but with PMSF, served as blank.

### Site direct mutagenesis

To confirm the predicted catalytic residues, Ser-294-Ala, Asp-391-Ala, and His-425-Ala mutants of Rv1288 were created by overlapping extension PCR. Primers used for the mutagenesis were mentioned in Table S1. Mutations were confirmed by gene sequencing. All the mutants were cloned, expressed, and purified according to the protocol used for wild type Rv1288 protein. Enzyme activities of mutants and wild type proteins were determined by standard lipolytic enzyme assay. Residual activity was calculated by taking enzyme activity of wild type protein as 100%.

### Biophysical characterization

For biophysical studies, CD spectra of both the proteins were obtained (JASCO J-815 spectrophotometer, Japan) at 190–260 nm wavelength using 2 mM path length cuvette at RT. The negative ellipticity was expressed as [θ], degcm^2^ dmol^−1^. Secondary structure elements were estimated quantitatively by spectra software using Yang's reference. Purified proteins (100 μg) were incubated at different temperature (20 to 90°C) for 10 min and spectra were collected at 190–260 nm wavelengths at 1 nm band width. Buffer served as blank for each spectrum.

To determine the intrinsic fluorescence, fluorescence spectra of both the proteins were obtained (JASCO J-815 spectro fluorometer, Japan) using 10 mm path length quartz cuvette at excitation wavelength 295 nm (5 nm slit width). Emission was recorded at wavelength 310–400 nm (10 nm slit width). The spectra of proteins pre-incubated (10 min) at different temperature (20 to 90°C) were also collected. Buffer served as blank for each spectrum.

### Subcellular localization of Rv1288

The mid log phase culture of *M. tb* H37Ra was harvested and fractionated. Bacterial cells were centrifuged at 10,000 g for 30 min at 4°C. Extracellular proteins present in culture supernatant were precipitated with 80% ammonium sulfate saturation and dialyzed against 1 X PBS and used as culture filtrate fraction. Cell pellet was washed twice with PBS, and further lysed by sonication in PBS (10 × of 60 s cycle with 2 min cooling period). Cell lysate were centrifuged twice at 4,000 × g for 10 min to remove the unlysed cells. Supernatent was further centrifuged at 27,000 × g for 1 h at 4°C to separate the cytosolic fractions (supernatant) and cell wall fractions (pellet) (Rezwan et al., 2007).

### Raising antibody against rRv1288

Purified rRv1288 was used to raise polyclonal antibodies in rabbit against the protein. Appropriate ethical clearance was taken from Institutional Ethics Committee, Panjab University, Chandigarh. Protein (200 μg) was emulsified with Freund's complete antigen (1:1 ratio) for first dose and with Freund's incomplete antigen (1:1 ratio) for two booster doses. Two weeks old rabbit were acclimatized in animal house conditions for 2 weeks. At day 1, blood was obtained from rabbit via orbital bleeding method (puncturing marginal ear vein) for control sample. At day 1, first dose was administered by subcutaneous injection. At day 14 and day 28, first and second booster doses were given, respectively. At day 33, blood sample was obtained and kept at 37°C for 1 h for the blood clotting. Blood was centrifuged at 4,000 rpm at 4°C for 15 min. The upper transparent layer of serum was carefully separated and stored at −20°C. Serum was checked for the presence of antibodies by dot blot experiment. Cross reactivity of Rv1288 IgG antibody was checked with the bacillus lipase, LipV and Lip N of *M. tuberculosis*.

The presence of Rv1288 protein in different fractions was identified by western blot using rabbit generated anti Rv1288 IgG (1:2,000) primary antibody, followed by alkaline phosphate conjugated anti rabbit IgG secondary antibody (1: 5,000) with intermittent washing. Anti-GroEL IgG (1:5,000) and Anti-antigen 85 complex (1:5,000) generated in rabbit were used as marker to check the purity of fractions. BCIP-NBT substrate (Chromus Biotech, India) was used to develop the blot.

### Peptidoglycan binding assay

Peptidoglycan binding assay was performed according to the modified protocol from (Yoshida et al., 1996). Briefly, 1 mg of *Bacillus* peptidoglycans (Sigma Co., USA) were dispersed in 1 ml of Tris binding buffer (20 mM Tris-Cl buffer, pH 6.8, and 150 mM NaCl). Peptidoglycans were washed twice with 1 ml of Tris binding buffer by centrifugation and sedimentation at 12,000 g for 5 min. The sedimented peptidoglycans were resuspended in 500 μl of Tris binding buffer. 100 μl of each protein (250 μg/ml) was mixed with 60 μl of Tris binding buffer and 160 μl of peptidoglycan suspension. Mixture was incubated at 4°C for 30 min and centrifuged for 5 min at 12,000 *g*. The peptidoglycan pellets were transferred to fresh microcentrifuge tubes and washed with 300 μl of Tris binding buffer. The pellet of purified rRv1288 without peptodoglycan was used as control to rule out the possibility of getting some denatured or aggregated protein in pellet. The supernatant and pellet fraction were subjected to SDS-PAGE.

### Cloning and expression of *rv1288, est*, and *lyt* in *M. smegmatis*

For cloning in *M. smegmatis*, all the recombinant pET28a plasmid (*rv1288, est, lyt*, and *S294A* mutant) and pVV16 *E. coli-Mycobacterium* shuttle vector were double digested with restriction enzymes (BamHI and HindIII). The digested gene fragments from pET28a were ligated with the digested pVV16 vector and used to transform *E. coli* DH5α cells. Transformants were screened for the presence of insert by colony PCR and plasmid migration, followed by confirmation by sequence analysis. Recombinant plasmids were electroporated into competent *M. smegmatis* mc^2^155 cells at 25 μF and 2.5 kV for 5 ms in Gene pulser, (BioRad, USA) followed by immediate addition of M7H9 media. After electroporation, cells were grown at 37°C, 180 rpm for 6 h, followed by spreading on M7H10 agar plates supplemented with kanamycin and hygromycin. Plates were incubated at 37°C for 5–6 days.

For localization, *MS_Est* and *MS_Lyt* (recombinant *M. smegmatis* strains cloned with Est and Lyt domain, respectively) were fractionated using same protocol as mentioned above. Western blot was performed using mice anti-His IgG (1:5,000) as primary antibody and alkaline phosphate conjugated anti mice IgG (1:5,000) as secondary antibody with intermittent washing.

### Colony morphology and growth kinetics of *MS_Vec* and *MS_1288*

*M. smegmatis* expressing *rv1288 (MS_1288)* and vector alone (*MS_Vec)* were spread on M7H10 agar plates supplemented with 1% glycerol, 10% OADC, and 0.05% Tween-80. Plates were incubated at 37°C for 5–6 days. For the high resolution, microphotography of the colonies was carried out by Nikon Camera. For growth kinetics *MS_Vec* and *MS_1288* were inoculated (from 1% overnight grown cultures) in 100 ml of M7H9 supplemented with 1% glycerol, 0.05% Tween-80, and 10% OADC and grown at 37°C, 180 rpm. Absorbance at 600 nm was measured and colony forming units (cfu) were determined at regular interval of 24 h.

### Mycobacterial cell surface properties: pellicle and aggregate formation

For pellicle formation all the cultures were grown in 10 ml M7H9 medium with 1% glycerol, 10% OADC (without Tween-80) at 37°C without shaking. Cultures were photographed at interval of 24 h (Singh et al., 2016b). For aggregate formation all the cultures were grown in 10 ml M7H9 medium with 1% glycerol, 10% OADC (without Tween-80) at 37°C, 180 rpm for 24 and 48 h. Cell aggregates formed in both the cultures were allowed to settle down by placing the tubes at static conditions at RT and then photographs were captured.

### Effect on drug resistance

*MS_Vec* and *MS_1288* were also checked for the antibiotic sensitivity using resazurin colorimetric redox indicator test (Kumar et al., 2017c). Both the cultures were grown to mid log phase. Equal number of cells (2 × 10^5^) were suspended in M7H9 media devoid of Tween-80 and added to the 48 well plates. Drug were diluted and added to the wells at different concentrations of rifampicin (1, 2, 4, 6, 8, 10 μg/ml), streptomycin (0.5, 1, 2, 4, 8, 16 μg/ml) and isoniazid (0.5, 1, 2, 4, 8, 16 μg/ml). Plates were incubated for 2 h at 37°C. Fresh working solution of resazurin was prepared by diluting the 10X stock (in 20% Tween-80) in 1:1 dilution. Eight microliterof working resazurin solution was added to each well. Plates were incubated at 37°C for 2 days. Plates were observed for change of blue to pink color at regular interval of time.

In another experiment, equal number of cells (2 × 10^5^) were suspended in M7H9 media devoid of Tween-80 and added to the 48 well plates. Drug were diluted and added to the wells at different concentrations of rifampicin (1, 2, 4, 6, 8, 10 μg/ml), streptomycin (0.5, 1, 2, 4, 8, 16 μg/ml) and, isoniazid (0.5, 1, 2, 4, 8, 16 μg/ml). Plates were incubated for 2 h at 37°C. Cells were appropriately diluted and spread on the M7H10 plates supplemented with kanamycin and hygromycin for cfu count.

### Lipid composition of cell wall of *MS_Vec* and *MS_1288*

Both the recombinant *M. smegmatis* strains were grown to mid log phase. Cells were harvested by centrifugation at 4,000 rpm for 10 min. Total lipids were extracted from equal weights of mycobacterial wet pellets, by continuous stirring in 50 ml chloroform-methanol (2:1, v/v) at RT for overnight. This organic phase was evaporated in rotary evaporator to obtain the whole cell lipids in dried form. Amount of lipid content for both the cultures was measured by weighing and equal amount of both the lipids were resuspended in 100 μl of chloroform. Two microliters of each lipids were loaded on Silica gel 60 plates (Merck, USA) for separation of different types of lipids including polar, apolar lipids, glycolipids and mycolic acid containing glycolipids by thin layer chromatography (TLC) analysis on using different mobile phases as mentioned in Table 1. TDM (Sigma, 10 mg/ml) was used as control in mycolic acid TLC plates. Iodine staining was used to visualize the TLC plates.

**Table 1:** Mobile phase composition of TLC for separation of different types of lipids.

**Type of lipids**	**Mobile phase used**	
	**Components**	**Ratios of components**
Polar lipids	Chloroform-Methanol-Water	65:25:4, vol/vol/vol
Apolar lipids	Hexane-Ethylacetate	90:10, vol/vol
Glycolipids	Methanol: Ammonium Hydroxide	80:20, vol/vol
Mycolic acid	Chloroform: Methanol: Ammonium Hydroxide	80:20:2 vol/vol/vol

### Effect of nutrition stress on survival of *MS_Vec* and *MS_1288*

*MS_1288* and *MS_Vec* were grown in M7H9 medium till mid log phase. Cells were centrifuged at 2,000 g for 5 min and washed twice with 1X PBS (pH 7.4). Cell pellets were resuspended in 1X PBS and were incubated at 37°C for 24 h. For determination of cfu count, cells were serial diluted and plated onto M7H10 agar plates supplemented with kanamycin and hygromycin.

### Effect on growth of *MS_1288* in presence of different lipids

Equal number of cells of *MS_1288* and *MS_Vec* were inoculated in 5 ml of different media[M7H9 medium supplemented with 1% glycerol, or 1% tributyrin (C4) or 1 mg/ml trilaurin (C12)]. All the tubes were incubated at 37°C, 180 rpm for 24 h. The cells growing on M7H9 media alone served as control. For determination of cfu count, cells were serial diluted and plated onto M7H10 agar plates supplemented with kanamycin and hygromycin.

### Effect on intracellular survival

Murine macrophage cell line RAW 264.7 were maintained in Dulbecco's Modified Eagle's Medium (DMEM) containing 10% FBS, streptomycin (100 μg/ml), penicillin (100 IU/ml) and 1% L- Glutamine at 37°C in CO_2_ incubator (5% CO_2_). For intracellular survival, cells were seeded in 12 well (2 × 10^5^ cell per well) surface treated culture plates in DMEM medium (without antibiotics). *MS_Vec* and *MS_1288* were internalized into macrophage cell line with MOI values 10:1 ratio for 3 h at 37°C in 5% CO_2_. Afterwards, media was removed from the wells. Cells were washed twice with PBS. DMEM media with antibiotics was added to kill the extracellular bacteria and then cells were incubated for 1, 6, 12, 24, 48, and 72 h. At each time interval, cells were again washed twice with PBS. Cells were lysed with 100 μl lysis buffer (0.1% triton X 100 in PBS). The numbers of intracellular mycobacteria were enumerated by spreading suitable dilutions onto the M7H10 agar plates containing Kan+/Hyg+ antibiotics, to compare the ability of cultures to infect macrophages.

## Results

### Up-regulation of *rv1288* expression in nutritive stress

**Figure 1:**
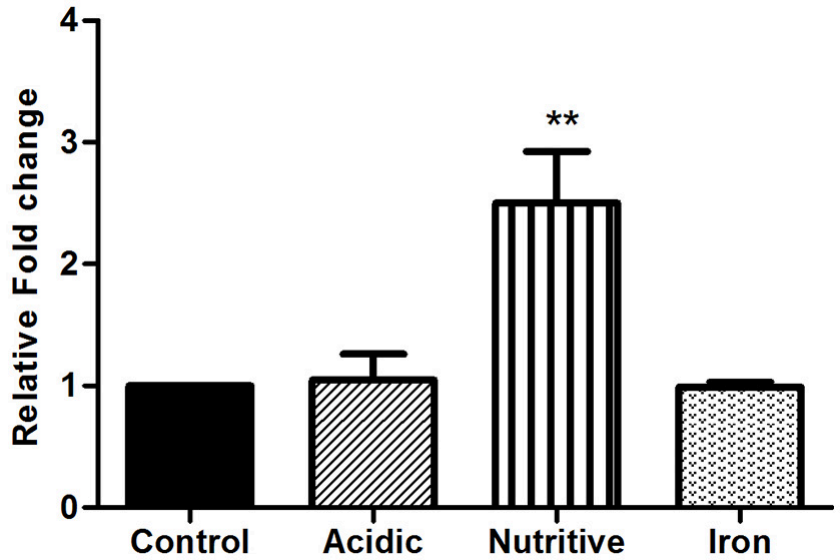
Expression analysis of *rv1288* under different stress conditions by real time PCR. The mid log phase culture of *M. tb* H37Ra culture was exposed to acidic, nutritive and iron stress for 6 h. Relative mRNA expression of *rv1288* was calculated with housekeeping gene *16S rRNA* as internal control. All experiments were carried out three times in triplicate (*n* = 3) and error bars indicated standard deviation. Student's *t*-test was performed to show the statistical significance (**p < 0.01).

Once inside the host, mycobacterium encountered various stress conditions. Expression of genes of mycobacteria changed during stress conditions (acidic, nutrient, and iron starvation)might help the adaptation and survival of bacilli inside host cells. Therefore, we investigated the expression of *rv1288* in *M.tb* H37Ra grown under different *in vitro* stress environment and it was found to be up-regulated by 2.5 fold under nutrient depletion condition with respect to *16S rRNA* reference gene. However, no significant change in expression of *rv1288* was observed under acidic and iron stress conditions (Figure 1).

### Comparative esterase activity of rRv1288 and its domains

**Figure 2:**
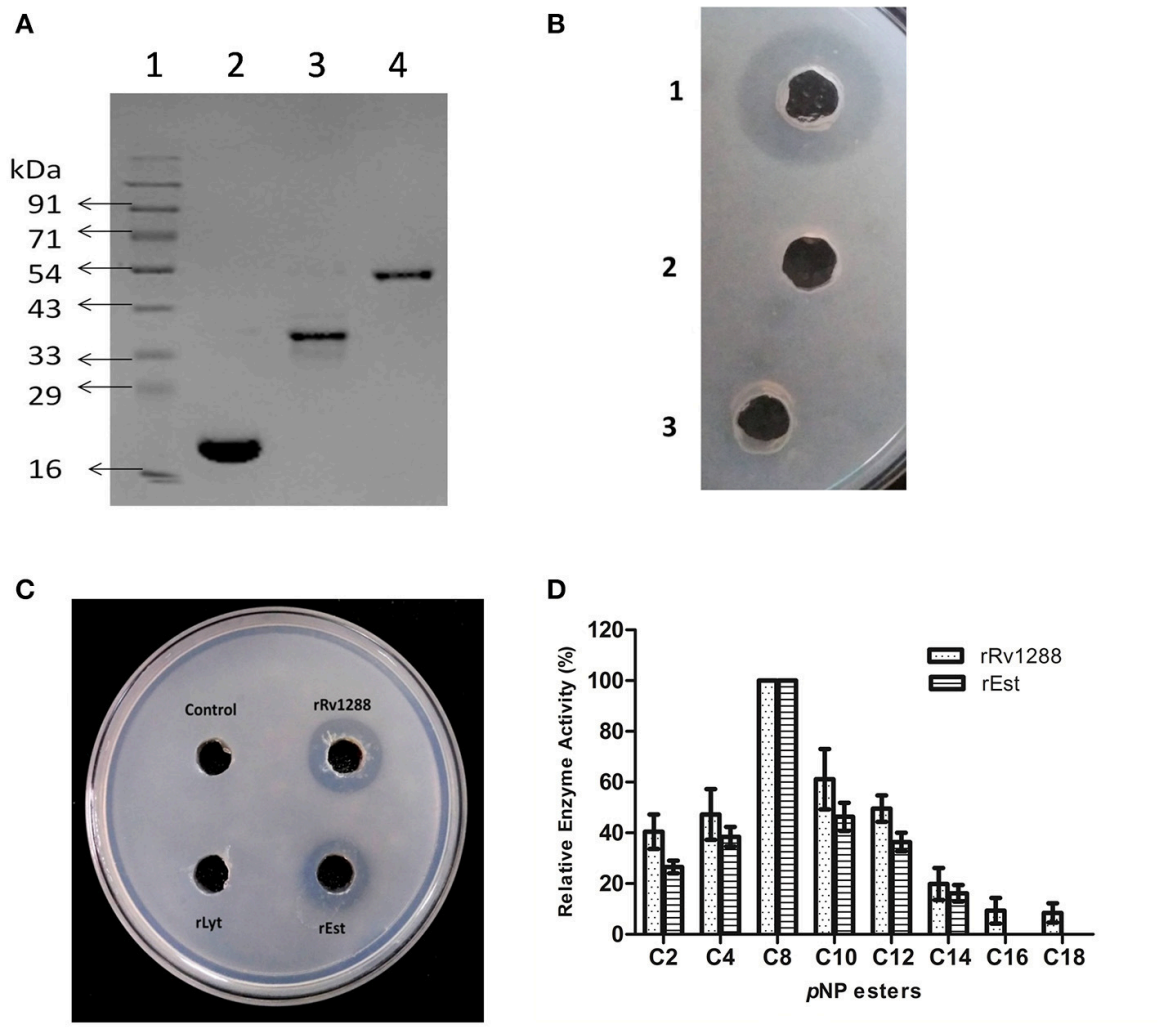
**(A)** SDS–PAGE analysis of purified rRv1288 and its domains. 1: Protein Marker, 2: Purified rLytE domain, 3: Purified esterase domain (rEst), 4: Purified full length Rv1288. **(B)** Skimmed milk plate for protease activity. 1 = Trypsin, 2 = 50 mM phosphate buffer and 3 = purified rRv1288. Trypsin was used as positive control. **(C)** Tributyrin plate assay of rRv1288 and its domains. 50 mM phosphate buffer was taken as control. **(D)** Substrate specificity of Rv1288 and Est domain toward different p-NP esters. C2: pNP-acetate, C4: pNP-butyrate, C8: pNP-caprylate, C10: pNP-capricate, C12: pNP-laurate, C14: pNP- myristate, C16: pNP-palmitate, C18: pNP- stearate. The enzyme activity with pNP-caprylate was taken as 100%. The values represent the means ± *SD* of three independent experiments.

Expressions of all the three proteins (rRv1288, rEst domain, and rLyt domain) were optimized to get the expressed proteins in the soluble fraction and were purified by affinity chromatography (Figure 2A). All the proteins were purified to homogeneity. The purified rRv1288 did not show any proteolytic activity as no zone of clearance was observed on skimmed milk plate (Figure 2B). However, purified rRv1288 and rEst proteins showed clearance zone on emulsified tributyrin plate (Figure 2C), while no zone was observed with rLyt domain. The lipolytic activity of enzyme was further investigated with synthetic lipolytic substrates, *p*NP esters of different carbon chain length. Both the rRv1288 and rEst proteins exhibited hydrolytic activity, with *p*NP-caprylate (C8) as optimum substrate followed by *p*NP- capricate (C10) while no lipolytic activity was observed with rLyt protein. Increasing carbon chain length beyond C14 drastically decreased the enzyme activity of both the proteins (Figure 2D). Specific activity of rRv1288 and rEst were 96 ± 8 and 92 ± 5 U/mg, respectively with *p*NP-caprylate as substrate.

### Effect of temperature and pH on activity and stability of rRv1288 and rEst proteins

**Figure 3:**
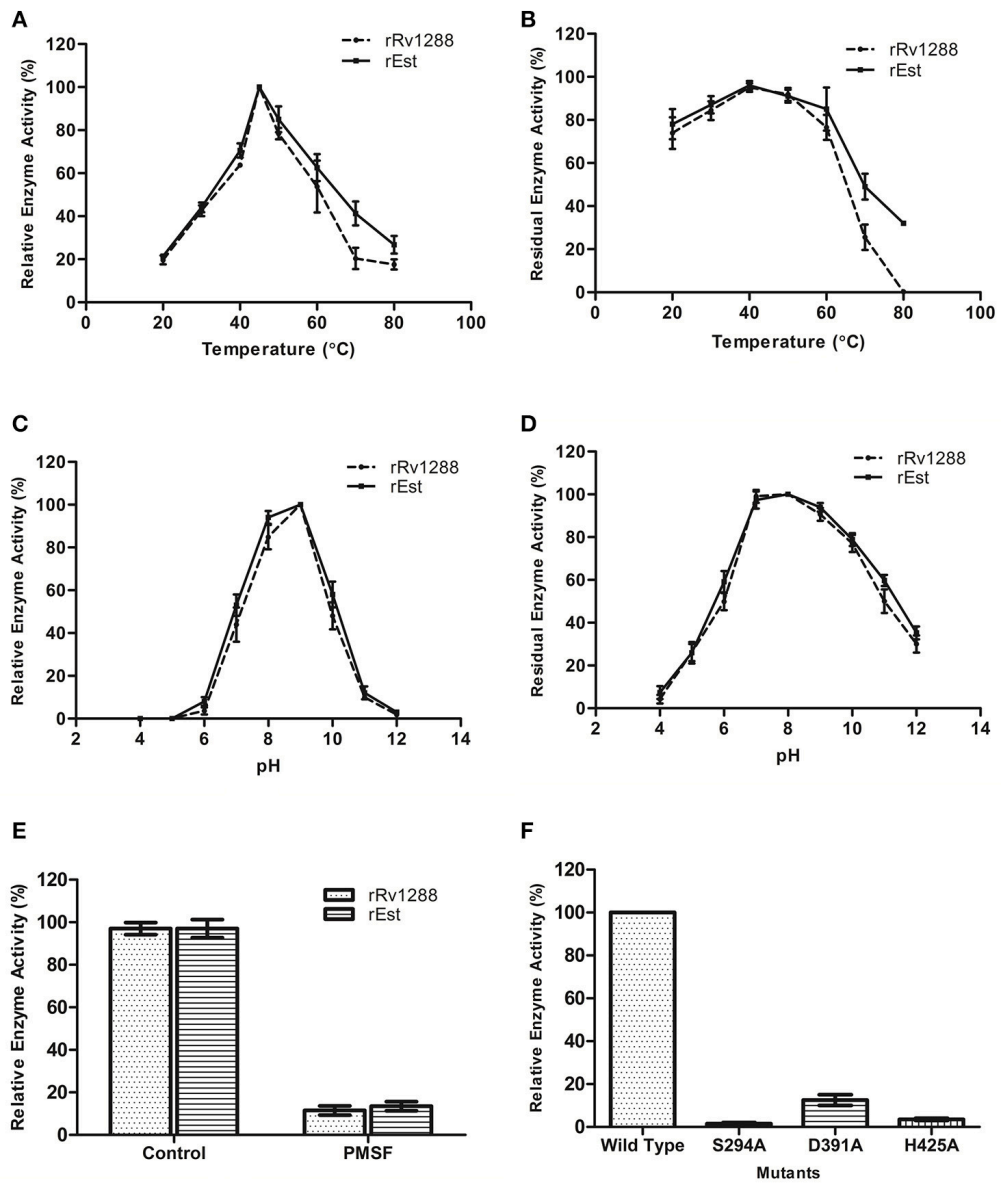
Biochemical characterization of rRv1288 and Est Domain. Effect of temperature **(A)** and pH **(C)** on enzyme activity. Effect of temperature and pH on enzyme stability **(B,D)** of rRv1288 (dashed line), and its Est domain (solid line). The relative enzyme activity were expressed as a percentage activity at pH 9.0 and 45°C. For checking stability, enzymes were incubated at respective pH and residual activity were calculated by taking the activity of purified enzymes (without incubation) as 100% in standard conditions. **(E)** Effect of PMSF on enzyme activity of rRv1288 and rEst proteins. **(F)** Relative enzyme activity of mutants (Ser294Ala, Asp391Ala, and His425Ala) in comparison to wild type Rv1288 protein. Results are representative of three independent biological replicates and shown as mean ± *SD*.

Optimum temperature and pH for activity of rRv1288 and rEst protein was observed to be 45°C and pH 9.0 (Figures 3A,C). Both the proteins displayed negligible activity and stability at higher temperature (70 and 80°C) and extreme pH (4.0, 5.0, 11.0, and 12.0) (Figures 3B,D). Thus, both the full length Rv1288 and esterase domain displayed similar biochemical characteristics. All parameters were almost similar for both the proteins suggesting that LytE domain did not alter the optimum temperature/pH for enzyme activity as well as stability of esterase domain.

### Kinetic studies

Kinetic studies of rRv1288 and rEst were performed at different concentration of substrate ranging from 0.1 to 1 mM. The apparent Michaelis- Menten constant (K_m_) and maximum velocity (V_max_), turnover number (*k*_cat_) and catalytic efficiency (*k*_*cat*_ / K_m_) were calculated from Lineweaver-Burk plot (Figure S1) and mentioned in Table 2. All parameters were almost similar for both the proteins suggesting that LytE domain did not alter the catalytic activity of full length protein.

**Table 2:** Kinetic parameters of recombinant rRv1288 and rEst protein.

	**app.*K_*m*_* (μM)**	**app.*V_*max*_*(μmoles min^−1^ml^−1^)**	**app.*k_*cat*_*(min^−1^)**	**app*.k_*cat*_/*app.*K_*m*_*(μM^−1^ min^−1^)**
rRv1288	77 ± 7	11 ± 3	2.0 ± 0.1	0.03
rEst	67 ± 5	7 ± 2	2.0 ±0.3	0.03

### Catalytic residues for enzyme activity

Significant loss in esterase enzyme activity (90%) was observed with Ser modifier, PMSF for both rRv1288 and rEst proteins (Figure 3E). To validate the predicted catalytic residues S294A, D391A and H425A mutants of Rv1288 were generated. Complete loss of enzyme activity was observed in S294A and D425A mutants. However, D391A mutant lost nearly 85% the total enzyme activity as compared to wild type rRv1288 protein (Figure 3F).

### Biophysical studies

**Figure 4:**
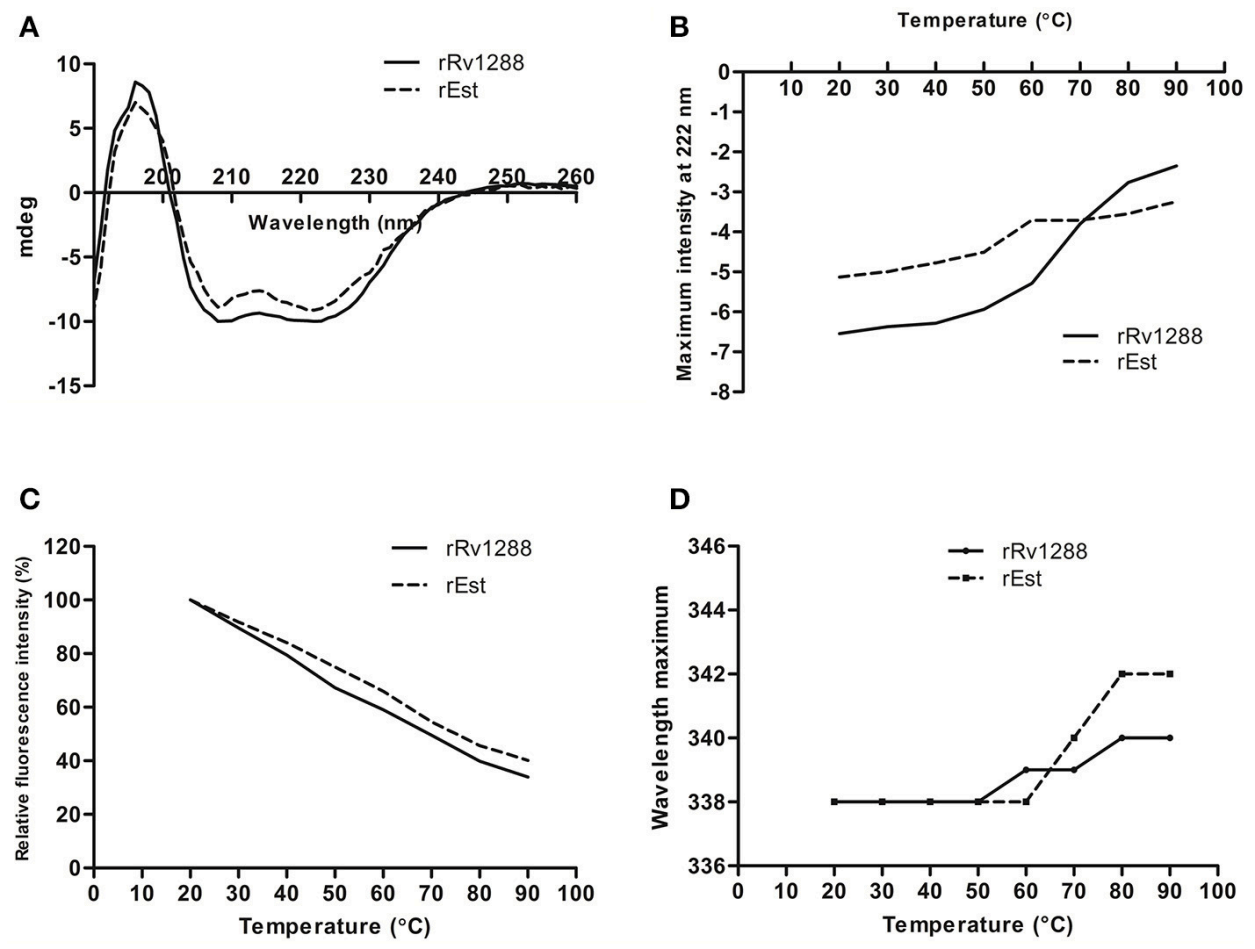
Biophysical Characterization of rRv1288 and rEst. **(A)** Far—UV CD spectra of recombinant purified rRv1288 and rEst proteins measured in 10 mM phosphate buffer (pH 8.0) at 25°C at wavelength 190–280 nm. **(B)** Thermal unfolding of rRv1288 and rEst proteins recorded at far UV-CD spectra at 222 nm wavelength with temperture increasing from 20 to 90°C at rate of 1°C/min. **(C)** Effect of temperature on maximum fluorescence intensity. Relative maximum fluorescence intensity of rRv1288 and rEst proteins with temperature increasing from 20 to 90°C. **(D)** Wavelength peak maxima for rRv1288 and rEst proteins at different temperatures (20 to 90°C). Each spectra represented the average data of three independent scans.

Presence of α/β hydrolase fold in rRv1288 and rEst proteins was confirmed by Far UV CD spectra (Figure 4A). The relative amount of secondary structure of rRv1288 was calculated to be 25% α-helix, 20.8% β-sheets, 25.4% turns, and 28.7% random coil, while, 21.4% α-helices, 25.4% β-sheets, 36.4% turns, and 16.8% random coil for rEst respectively. Both recombinant proteins demonstrated similar CD spectra pattern, with higher minima at 208 nm than at 222 nm. During the thermal unfolding studies of both the proteins, negative molar ellipticity reduced gradually with rise in temperature (Figure 4B). The helical conformation and minima at 222 nm of proteins were stable up to 50°C. Beyond this the minima was abruptly shifted, indicating the unfolding/ denaturation of protein.

Effect of temperature on conformation change in tertiary structure was also monitored by measuring the intrinsic tryptophan fluorescence intensity. The rRv1288 and rEst protein has 9 and 8 tryptophan amino acid, respectively. The maximum intensity was recorded at 338 nm for both enzymes. With rise in temperature, fluorescence intensity was gradually decreased. Rate of reduction in maximum peak intensity is slightly more in rRv1288 as compared to rEst. Peak maxima were also shifted from 338 to 340 nm and 342 nm for rRv1288 and rEst respectively, with increase in temperature from 50 to 90°C (Figures 4C,D). Shifting of peak maxima toward higher wavelength is known as red shift. So, higher red shift was observed in case of rEst. Buffer was used as blank for each spectrum.

### Localization

**Figure 5:**
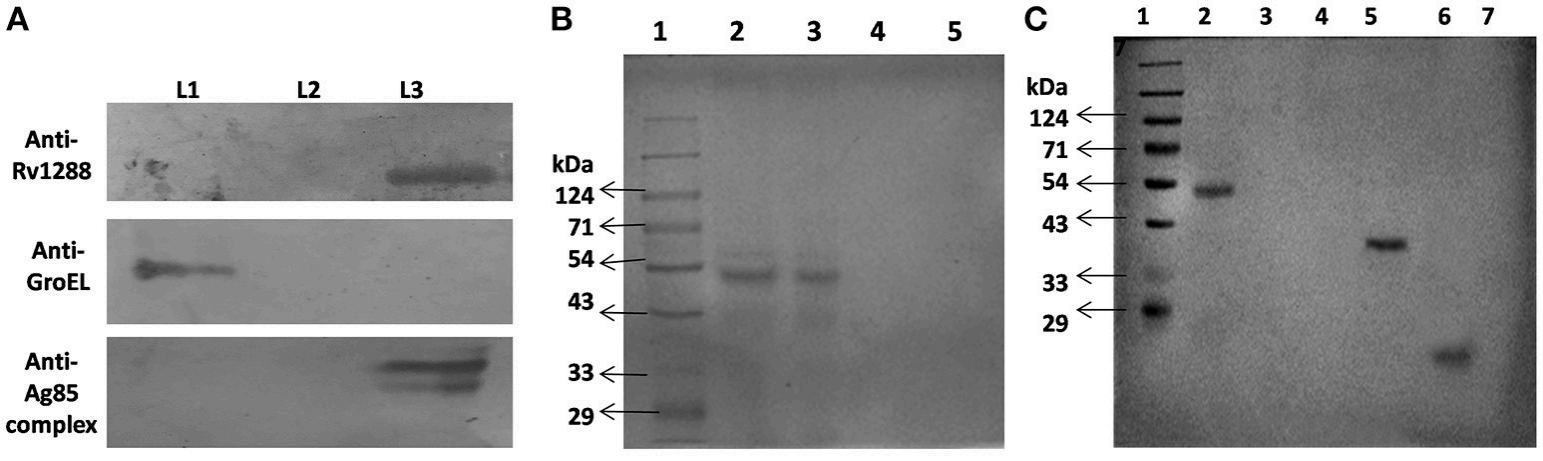
**(A)** Immunolocalization of Rv1288, GroEL and antigen 85 complex in *M. tb* H37Ra. L1 = Cytosolic fraction, L2 = Culture filtrate protein fraction, L3 = Cell wall fraction. **(B)** Peptidoglycan Binding Assay of rRv1288. 1: Protein marker, 2: Purified rRv1288 protein, 3: Pellet fraction after incubation of rRv1288 and peptidoglycan, 4: Supernatant fraction after incubation of rRv1288 and peptidoglycan, 5: Pellet fraction of control (only protein, no peptidoglycan). **(C)** Peptidoglycan Binding Assay of rRv1288 and its domains. *Bacillus* peptidoglycans were incubated with the purified rRv1288, Est and Lyt proteins for 30 min, centrifuged, washed and loaded on the SDS-PAGE gel. 1: Protein Marker, 2: Pellet fraction after incubation with rRv1288, 3: Supernatant fraction after incubation with rRv1288, 4: Pellet fraction after incubation with rEst protein, 5: Supernatant fraction after incubation with rEst protein, 6: Pellet fraction after incubation with rLyt protein, 7: Supernatant fraction after incubation with rLyt protein.

For detection of localization of Rv1288, western blot analysis of cytosolic, membrane and secreted fractions of *M.tb* H37Ra was carried out using anti-Rv1288 polyclonal antibody raised in rabbit. Control marker GroEL and antigen 85 complex were observed in cytoplasmic fraction and membrane fraction, respectively, which indicated the purity of fractions. A 49 kDa band was observed only in the cell wall fraction, but not in the cytosolic and culture filtrate fractions (Figure 5A). This showed that Rv1288 is mainly present in cell wall fraction.

### Peptidoglycan binding assay

Full length rRv1288, rEst and rLyt domain were examined for their peptidoglycan binding capability. After incubating all the three proteins with peptidoglycan, only the full length rRv1288 and rLyt domain were detected in the pellet fraction containing peptidoglycans, while rEst domain was present in the supernatant fraction (Figures 5B,C). This indicated the rRv1288 could bind to peptidoglycan of cell wall and Lyt domain mediated this binding, while rEst domain did not have the binding affinity for bacillus peptidoglycans. However, no band was observed in the control, proving that pelting of proteins were peptidoglycan dependent and not due to insoluble proteins (Figure 5B).

### Effect of recombinant rv1288 over-expression in *M. smegmatis*

**Figure 6:**
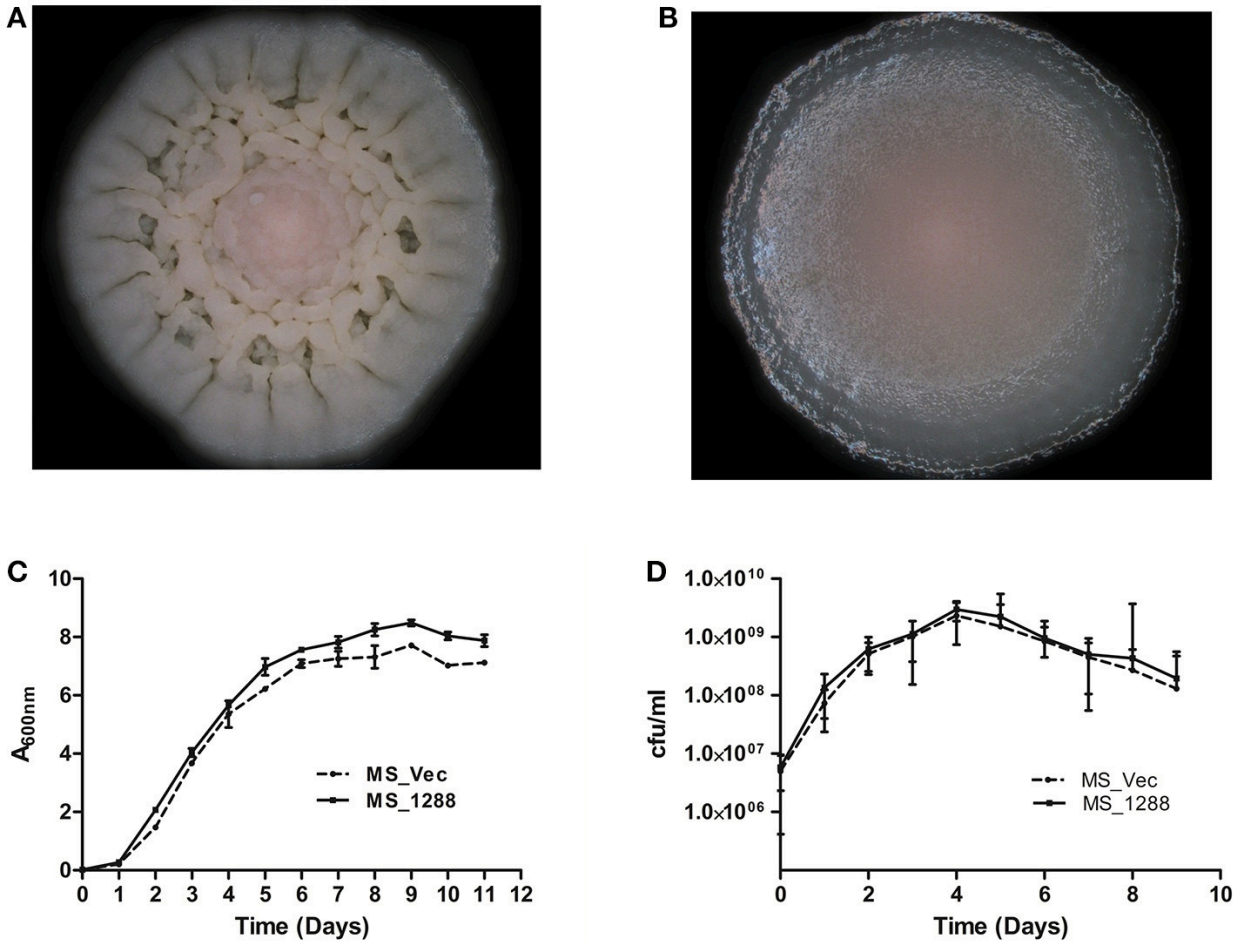
Effect of *rv1288* on Colony morphology: Enlarged view of single colony of **(A)** *MS_Vec* and **(B)** *MS_1288*. Graphs showing growth curve in **(C)** absorbance at 600 nm and **(D)** cfu count of *MS_1288* with *MS_Vec* as control. The data is representative of 3 independent experiments (mean ± *SD*).

To study the effect of over-expression of Rv1288 in *M. smegmatis, rv1288* gene was cloned in *E. coli- Mycobacterium* shuttle vector pVV16 and expressed in the surrogate host *M. smegmatis* mc^2^155. The colony morphology of *MS_1288* was different from the control *MS_Vec*. The colony surface of *MS_Vec* was dry, irregular, rough with bulging while for recombinant it was spherical, smooth and wet (Figures 6A,B). However, over-expression of *rv1288* did not alter the growth pattern of *M. smegmatis* mc^2^155 significantly (Figures 6C,D).

### Effect on membrane hydrophobicity

**Figure 7:**
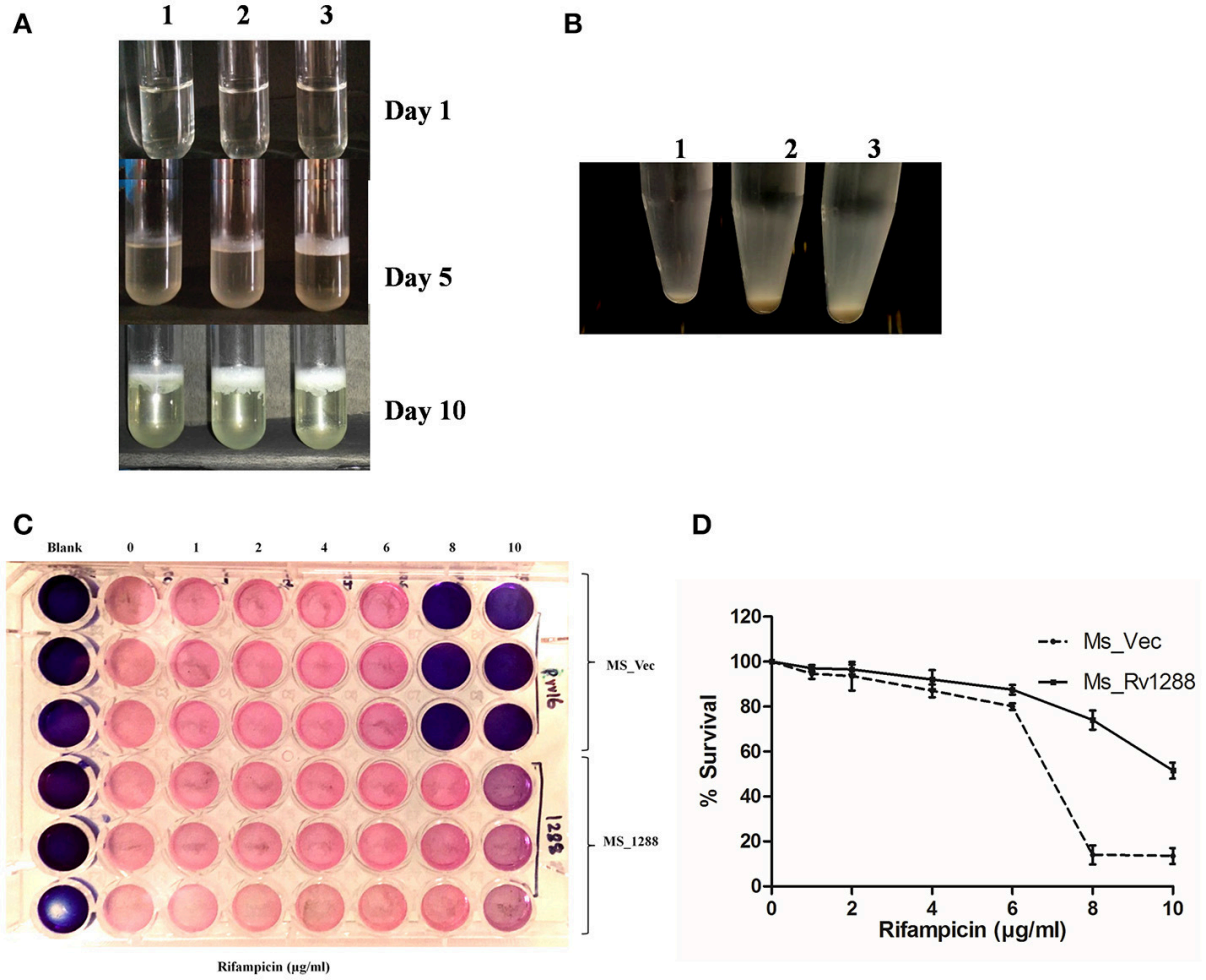
**(A)** Pellicle formation in standing cultures of *MS_Vec* (1), *MS_S294A_1288* (2) and *MS_1288* (3) (3) in M7H9 medium, in absence of Tween-80 at 37°C for various time points. **(B)** Aggregate formation by *MS_Vec* (1), *MS_1288* (2) and *MS_S294A_1288* (3) strains in M7H9 medium, absence of Tween-80 at 37°C with shaking for 48 h. Cultures were allowed to settle at room temperature for 30 min before taking photographs. **(C)** Effect of Rv1288 on drug susceptibility of *M. smegmatis*. Mid log phase culture of *MS_Vec* and *MS_1288* were diluted in M7H9 medium without Tween-80 and were treated with different concentrations of Rifampicin (1–10 μg/ml). After 2 h incubation resazurin dye was added and plates were observed for the change in color. Blue color represents dead bacterial cells and pink represents live cells. **(D)** *MS_Vec* and *MS_1288* were cultured in the presence of rifampicin at 37°C for 48 h. Survival of both the culture was measured by cfu count. Graph represent the % survival (Cfu count in the absence of rifampicin was considered as 100% survival). Experiment was performed in triplicate and results were shown as mean ± *SD*.

We also investigated the cell surface properties such as pellicle formation and cellular aggregation. Pellicles are biofilm like layer that formed at the air liquid interface in static cultures. *MS_1288* and formed pellicle earlier and thicker as compared to *MS_Vec* and *MS_S294A _1288* (Figure 7A) under similar experimental conditions. Aggregate formation in cultures was also examined in the absence of Tween-80. Tween-80 is a detergent that prevented clumping of mycobacterial cells. In absence of Tween-80, cellular aggregation resulted in reduced turbidity of cultures at 48 h. More aggregation was observed in *MS_1288* and *MS_S294A _1288* cells as compared to *MS_Vec* (Figure 7B).

### Effect on drug resistance

The effect of over expression of *rv1288* on drug sensitivity of *M. smegmatis* was studied in the presence of anti-TB drugs. *MS_1288* showed significant increase in drug resistance as compared to control *MS_Vec* culture in case of rifampicin. *MS_1288* exhibited resistance at 8 μg/ml of rifampicin, while control cells were killed at this concentration (Figures 7C,D). However, no significant change in drug sensitivity was observed in case of streptomycin and isoniazid (Data not shown). Enhanced drug resistance might be due to modulation of cell wall lipids/increased hydrophobicity of membrane and hence increase in permeability for rifampicin (hydrophobic molecule) but reduced permeability for hydrophilic isoniazid.

### Lipid content of *MS_Vec* and *MS_1288* cultures

**Figure 8:**
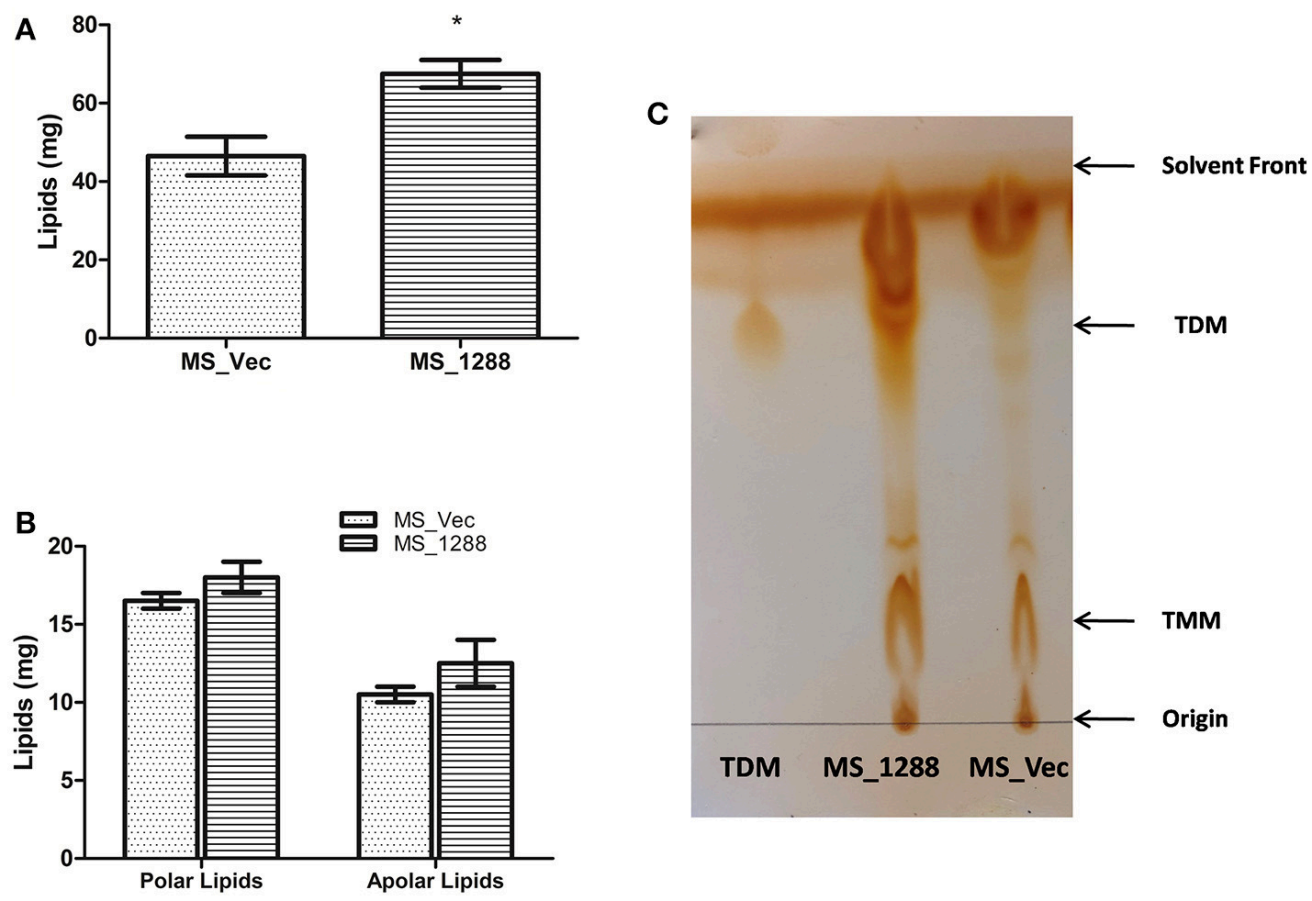
**(A)** Quantitative estimation of total lipids extracted from the equal weight of *MS_Vec* and *MS_1288*. **(B)** Polar and apolar lipids extracted from the equal weight of *MS_Vec* and *MS_1288*. **(C)** Mycolic acid containing glycolipids were separated from the pool of total polar lipids on TLC plate using chloroform: methanol: ammonium hydroxide (80:20:2) as mobile phase. TLC results showed the increased amount of TDM content in *MS_1288* in comparison to *MS_Vec*. Results are expressed as mean ± *SD* from three independent experiments. All experiments were carried out three times in triplicate (*n* = 3) and error bars indicated standard deviation. Student's *t*-test was performed to show the statistical significance (*p < 0.05).

Since the change in morphology, pellicle and aggregate formation as well as drug sensitivity was observed in previous experiments, we investigated the lipid content of *MS_Vec* and *MS_1288*. *MS_1288* had higher total lipid content as compared to control (Figure 8A). However, no difference was observed in polar and apolar lipid content (Figure 8B). Since mycolic acid is the main component of cell wall and Rv1288 was predicted to be a mycolyl acyl transferase, we further examined the mycolic acid rich glycolipids. TLC results showed the increased amount of TDM (Trehalose dimycolate) in *MS_1288* in comparison to *MS_Vec* (Figure 8C).

### Effect of nutrition stress on survival of *MS_Vec* and *MS_1288*

**Figure 9:**
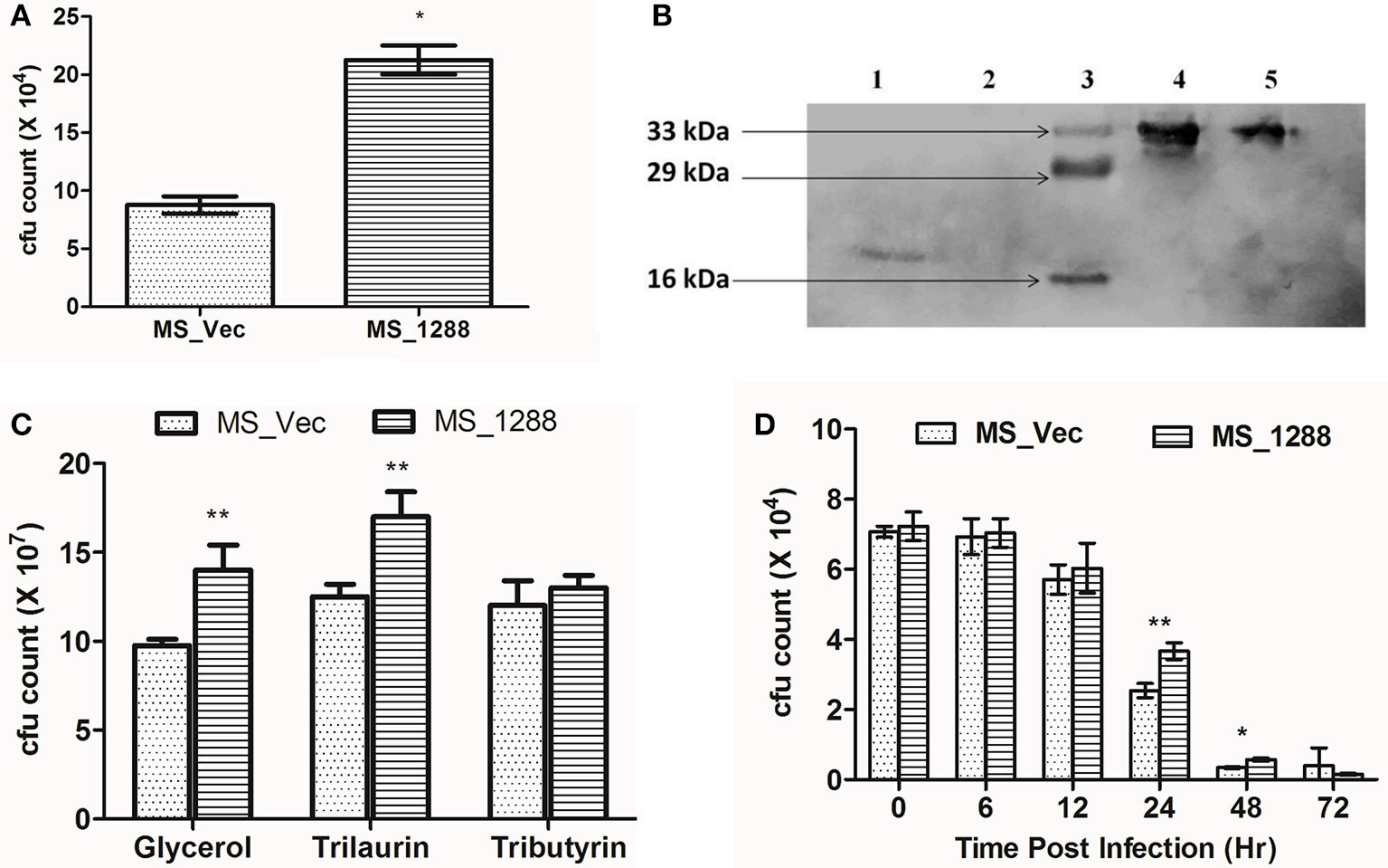
**(A)** Effect of expression of *rv1288* on survival of *M. smegmatis* in nutrition stress. Graph showing cfu count of *MS_Vec* with *MS_1288* under nutrition stressed conditions. The data is representative of 3 independent experiments. All experiments were carried out three times in triplicate (*n* = 3) and error bars indicated standard deviation. Student's t-test was performed to show the statistical significance (*p < 0.05). **(B)** Immunolocalization of Est and Lyt in recombinant *M. smegmatis*. 1 = Cell wall fraction of *MS_Lyt*, 2 = Cytosolic fraction *MS_Lyt*, 3 = Protein Marker, 4 = Cytosolic fraction *MS_est*, 5 = Cell wall fraction *MS_est*. **(C)** Cfu count of *MS_Vec* and *MS_1288* in presence of M7H9 medium supplemented with Glycerol, Trilaurin and tributyrin, at 37°C, 180 rpm for 24 h. **(D)** Intracellular survival of *MS_Vec* and *MS_1288*. Cfu count of intracellular *MS_Vec* and *MS_1288* were measured after 6,12, 24, 48, and 72 h post infection. The data is representive of three independent experiments.

Since *rv1288* was found to be upregulated under nutrition starvation conditions in *M. tuberculosis* H37Ra, we also investigated the effect of same on survival of *MS_Vec* and *MS_1288* under nutrition starvation conditions*. MS_1288* exhibited nearly 3 folds higher cfu count (Figure 9A) under nutrition depletion condition than *MS_Vec*.

### Intracellular localization of Est and Lyt domain in *M. smegmatis*

Since, we found that Rv1288 protein was present in cell wall fraction of *M.tb* H37Ra, attempts were made to find out the localization of individual domain in recombinant *M. smegmatis* (*MS_Est* and *MS_Lyt*) using Anti-His antibody. Est domain was localized in both the cell wall and cytoplasmic fraction, while Lyt domain was found only in the cell wall fraction (Figure 9B).

### Effect on growth of *MS_1288* in presence of different lipids

Since Rv1288 was found to be membrane bound esterase, we tested the hydrolyzing efficiency of Rv1288 protein on lipids present in extracellular environment. *MS_Vec* and *MS_1288* culture were grown in the presence of glycerol, tributyrin and trilaurin instead of usual Tween-80 in the medium. *MS_1288* clearly showed the enhanced growth in the presence of glycerol and trilaurin as compared to control (Figure 9C). However, no significant change in growth was observed in case of tributyrin.

### Effect on intracellular survival

Inside the host, *M. tb* resides mostly in macrophage cells and they are commonly used as model system to study the intracellular survival. Therefore, we tested the effect of *MS_Vec* and *MS_1288* in intracellular survival of *M. smegmatis* in Raw 264.7 murine macrophage cell lines. Expression of Rv1288 enhanced the survival of *M. smegmatis* inside the macrophage cell line at 24 h and 48 h (Figure 9D).

## Discussion

The mycobacterial proteins categorized as conserved, hypothetical with unknown functions restricted our understanding of physiology of this pathogen. Several hypothetical proteins shared characteristic feature with the other functionally known proteins. Many of these proteins including Rv1288 consisted of α/β hydrolase fold, i.e., characteristic features of lipase/esterase/protease enzymes and predicted to have hydrolase activity (Kumar et al., 2017d). Other than Rv1288, only one protein, Rv2719, a cell wall hydrolase and potential regulator of cell division in *M.tb*, was reported to have Lyt domain (Chauhan et al., 2006). Earlier reports suggested that proteins having LysM domain act as virulence factor in several human pathogens like *Staphylococcus aureus, Listeria monocytogenes, Neisseria meningitides, Francisella tularensis, Streptococcus agalactiae* etc. (Martin et al., 2002; Lenz et al., 2003; Kajimura et al., 2005; Melillo et al., 2006; Oldfield et al., 2007; Buist et al., 2008). Several lipases/esterases from mycobacteria, when expressed in *E. coli*, were reported to be expressed as inactive protein and formed inclusion bodies. However, absence of cysteine residue and disulfide bond along with aliphatic and instability index (< 40), predicted the expression of all the three proteins (Rv1288, Est, and Lyt) in soluble form (Idicula-Thomas and Balaji, 2005). Our study was in line with the predictions, as we were able to purify these proteins from the soluble fraction at low IPTG concentration and induction temperature. This strategy was successfully used for expression of proteins in soluble fraction in several other studies (Sinha et al., 2005; Zhang et al., 2005; Deb et al., 2006; Kaur et al., 2017b; Lin et al., 2017).

Conserved domain analysis revealed that Rv1288 belongs to “α/β hydrolase family,” which was further validated by CD spectroscopy. In contrast to the prediction by tuberculist, the protein demonstrated esterase activity and did not show protease activity. Both the full length and Est domain proteins showed similar specific activity and hydrolysis pattern, suggesting that Lyt domain did not affect the catalytic ability of Est domain. Our data was consistent with previous studies that demonstrated that several mycobacterium lipolytic enzymes prefer medium chain length esters (Guo et al., 2010; Sultana et al., 2013; Singh et al., 2014b) and short chain length esters (Zhang et al., 2005; Rengarajan et al., 2008; Shen et al., 2012; Jadeja et al., 2016; Singh et al., 2016a; Kumar et al., 2017e; Lin et al., 2017). The aggregate formation at higher temperature by rRv1288 only followed by loss in activity suggested the role of highly hydrophobic Lyt domain in protein aggregation and decreased stability of Rv1288. The protein aggregation during thermal unfolding was reported to contribute toward the smaller magnitude of red shift (Duy and Fitter, 2006). This study was in contrast to Rv0774c, where C terminal lipolytic domain is comparatively less stable than the full length protein (Kumar et al., 2017e). Previously, some mycobacterial lipases were reported to be stable at higher temperatures i.e., LipD (Singh et al., 2014a), LipV (Singh et al., 2014b),LipL (Singh et al., 2016a), LipN (Jadeja et al., 2016), and LipU (Kaur et al., 2017a).

Lyt domain is known to interact with the peptidoglycan moiety of cell wall (Buist et al., 2008). Molecular docking studies also demonstrated that it could bind to the N-acetyl glucosamine moiety of peptidoglycan (Kumar et al., 2017a). The peptidoglycan binding assay suggested that Rv1288 was attached to the cell wall of *M.tb* by Lyt domain. In line to our prediction, sub-cellular localization studies confirmed the presence of Rv1288 in cell wall fraction of *M.tb* H37Ra. This is consistent with mass spectrometry analysis that found the Rv1288 in membrane fraction of *M.tb* H37Rv (De Souza et al., 2011). Similar to our finding, Rv2719 protein of *M.tb*, containing Lyt domain was also localized on cell wall (Chauhan et al., 2006). Interestingly, in *M.tb* there are several cell wall anchored lipase/esterase such as LipY (Singh et al., 2014c), Rv0183 (Xu et al., 2010), and Rv2224c (Lun and Bishai, 2007) that were reported to play an important role in pathogenesis and virulence.

The proteome of *M.tb* is dynamic and kept changing in order to facilitate the survival of pathogen under stress conditions (Geiman et al., 2006; Saviola, 2010). Rv1288 was previously reported in hypoxia sample by mass spectrometry (James et al., 2013) and in dormant phase (Hasan et al., 2006; Murphy and Brown, 2007; Agüero et al., 2008). Current regimens are not much effective against the bacterium in latent phase, especially in hypoxic granulomas present in the lung (Murphy and Brown, 2007). It was earlier reported that survival of the mycobacteria depend on their potential to metabolize the stored lipids, as they are the major energy source during latency (Barisch and Soldati, 2017). In this study, *rv1288* was up-regulated under nutrient starved condition and expression of *rv1288* gene in *M. smegmatis* resulted in enhanced survival under nutrient starved condition. As this is a membrane bound enzyme and it showed enhanced growth in presence of glycerol and trilaurin as carbon source, the probable role of this protein in host lipid utilization cannot be ruled out. Thus, it was suggested that under nutrient starved condition, Rv1288 might hydrolyze intracellular or extracellular lipids in order to facilitate its survival. Previously, other lipases LipY and LipX were also reported to be up regulated in nutrition starvation (Betts et al., 2002; Deb et al., 2006).

To mimic the over expression of *rv1288* in *M.tb* under stress condition, we over-expressed *rv1288* in *M. smegmatis*. Over expression of this protein resulted in altered colony morphology and slight increase in growth rate of bacterium. Similar to these results, expression of genes such as *rv1818c* (Delogu et al., 2004), *rv1169c* (Singh et al., 2016b) and *rv0774c* (Kumar et al., 2017c), changed the colony morphology and growth rate of *M. smegmatis*. It has been reported that colony morphology of mycobacterium species is associated with their virulence, drug resistance, infection, intracellular survival, immune modulation, and altered signaling pathways (Kuze and Uchihira, 1984; Shiratsuchi et al., 1993; Hubert et al., 2002). TLC results suggested that the Rv1288 might be responsible for increased amount of TDM content. The change in cell surface properties of *M. smegmatis* expressing *rv1288*, like colony morphology, pellicle formation, aggregation, and change in drug sensitivity pointed toward the remodeling of cell envelop resulting in change in permeability that might help the intracellular survival of mycobacteria. *MS_1288* also showed enhanced intracellular survival in Raw 264.7 cells as compared to the control. Various report showed that altered colony morphology, increase pellicle formation, and aggregative nature were attributed to the change in composition and remodeling of cell wall lipids (Singh et al., 2016b; Kumar et al., 2017c). In *M.tb*, mycolyl transferases are differentially expressed under different conditions to enable the bacteria to evade host immune system and survive (Ojha et al., 2010; Queiroz and Riley, 2017). Earlier, Takayama et al. (2005) reported Rv1288 as a probable mycolyl transferase II on the basis of its similarity to Ag85 complex proteins. Ag85 complex proteins catalyze the synthesis of TDM or cell wall arabinogalactan-mycolate from TMM by mycoyltransferase activity (Queiroz and Riley, 2017). Lipid profiling studies showed increase in total lipid and TDM content in *rv1288* expressing *M. smegmatis* strain. Virulent *M.tb* strains are usually rich in TDM (Singh et al., 2017). Similarly the expression of mycolyl transferase Ag85A protein in *M. smegmatis* resulted in increase concentration of the glycolipids TDM and TMM in the cell (Elamin et al., 2011). Almost all mycolyl transferases possessed carboxylesterase domain, and their ability to transfer the mycolyl moiety is usually dependent on this domain (Dautin et al., 2017). Recently, Rv0774c was also reported as mycolyl transferase II, that altered the lipid composition by synthesizing TDM (Kumar et al., 2017c). Rv1169c was also demonstrated to modulate the cell wall lipids (Singh et al., 2016b). Similar to our study, Rv3451 in *M.tb* was reported as TDM hydrolase, which was overexpressing under nutrition starvation conditions. Rv3451 was also reported to increase the growth rate and reactivation of *M.tb* in an immunocompromised animal model (Yang et al., 2014). These findings, strongly suggested that Rv1288 might possess mycolyl transferase activity.

## Conclusion

In summary, Rv1288 was confirmed as a cell wall anchored esterase of *M.tb* that was up regulated under nutrition depletion conditions and observed to support the bacterial growth under nutritive starvation condition. The Lyt domain was essential for placing the protein at cell wall. The expression of protein altered the colony morphology, cell surface properties, drug resistance, lipid content of the cell wall of *M. smegmatis* and its intracellular survival inside the macrophage cell line. The mycolyl transferase activity of Rv1288 could be correlated with increased concentration of the glycolipid TDM. These results pointed toward its important role in intracellular survival of mycobacteria. Further studies with knockout of *rv1288* in *M.tb*H37Rv are required to confirm the precise role of Rv1288 in lipid modulation.

## Authors contributions

PM performed the experiments and wrote manuscript. AK analyzed the data and manuscript writing. JasK performed experiments related to lipids. JagK conceived and designed the whole study.

### Conflict of interest statement

The authors declare that the research was conducted in the absence of any commercial or financial relationships that could be construed as a potential conflict of interest.
