# Supplementary material for: Rv1288, a Two Domain, Cell Wall Anchored, Nutrient Stress Inducible Carboxyl-Esterase of Mycobacterium tuberculosis, Modulates Cell Wall Lipid
Source: Front Cell Infect Microbiol. 2018 Dec 3;8:421. doi: 10.3389/fcimb.2018.00421 (PMC6287010; doi:10.3389/fcimb.2018.00421)
Supplement: Supplementary file 1 [file Data_Sheet_1.docx]

**Rv1288, a two domain, cell wall anchored, nutrient stress inducible carboxyl-esterase of *Mycobacterium tuberculosis*, modulates cell wall lipid**

**Maan Pratibha^1^, Kumar Arbind^1^, Kaur Jashandeep^1^, Kaur Jagdeep^1*^**

**Supplementary Data**

**Table S1:** List of primers used in the study.

| Gene | Primer Pairs | Annealing (°C) |
| --- | --- | --- |
| Rv1288 Lyt | FWD-5’ GGATCCATGGTCAGCACACATGCGGTT 3’  REV-5’AAGCTTGAATATGACCAGTACCTGCCCG 3’ | 63 |
| Rv1288 Est | FWD-5’ GGATCCATCGGGCGTAGCGACGGGTT 3’  REV- 5’ AAGCTTCTTATCGGCGCGTTCTGCGGC 3’ | 63 |
| Ser294 Ala | FWD- 5’ GCC GGG TTT GCG ATG GGT GGC 3’  REV-5’ GCC ACC CAT CGC AAA CCC GGC 3’ | 63 |
| Asp391Ala | FWD- 5’ GCC AAC TGG TTC GCA AGC GTG AAC GA 3’  REV-5’ TC GTT CAC GCT TGC GAA CCA GTT GGC 3’ | 63 |
| His425Ala | FWD-5’ CCT GGC GGT GCC GTC TTC CG 3’  REV-5’ CG GAA GAC GGC ACC GCC AGG 3’ | 63 |
| Sig A  (Internal Control) | FWD-5’CGATGACGACGAGGAGATCGC 3'  REV-5’ CAGCGCTACCTTGCGGATCTG 3' | 55 |
| Rv1288  Stress | FWD-5’ CTGGGAGACATTCCACATCG 3'  REV-5’ AAGTGGCCGTAGTACTTTGC 3' | 55 |


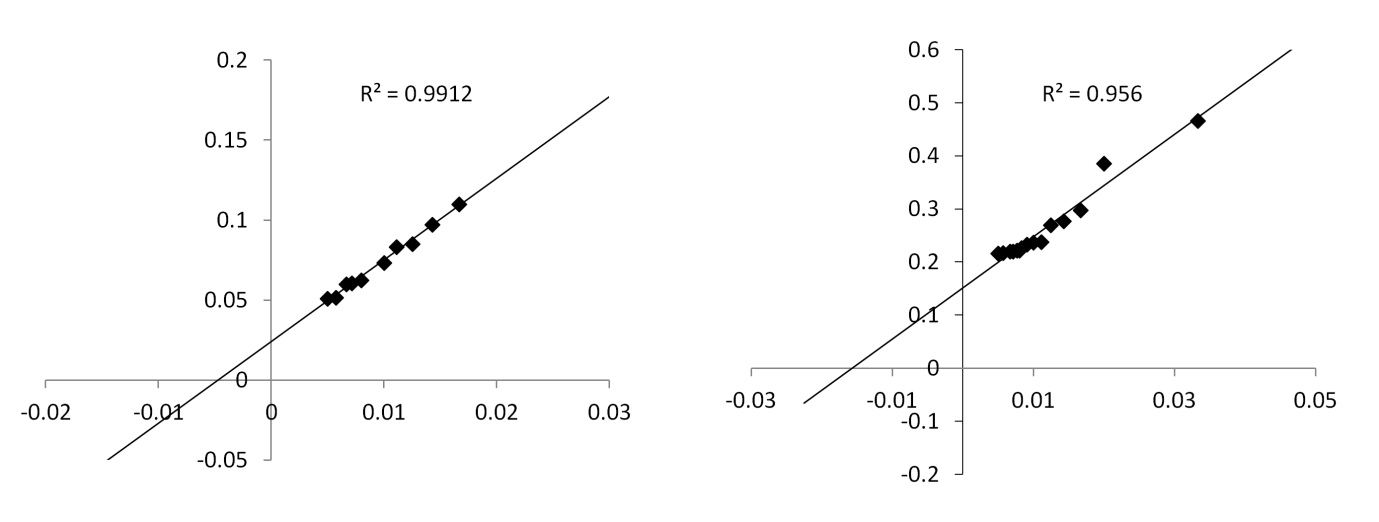


**(A) (B)**

**Figure S1:** Double reciprocal (Lineweaver–Burk) plot of rRv1288 **(A)** and rEst **(B)** proteins with the substrate *p*NP-octanoate. Each dot represents the mean value of three different measurements.
